# Synthesis of Novel Ultraviolet Absorbers and Preparation and Field Application of Anti-Ultraviolet Aging PBAT/UVA Films

**DOI:** 10.3390/polym14071434

**Published:** 2022-03-31

**Authors:** Run-Meng Qiao, Chi-Peng Zhao, Jia-Lei Liu, Mao-Lin Zhang, Wen-Qing He

**Affiliations:** Key Laboratory of Agricultural Film Pollution Prevention and Control, Ministry of Agriculture and Rural, Institute of Environment and Sustainable Development in Agriculture, Chinese Academy of Agricultural Sciences, Beijing 100081, China; xiaoqiaoya1123@163.com (R.-M.Q.); zhaochipeng@126.com (C.-P.Z.); zhangmaolin@mail.ipc.ac.cn (M.-L.Z.)

**Keywords:** poly-(butylene adipate-co-terephthalate), biodegradable film, tropolone, UV absorber, aging resistance, photostability

## Abstract

Poly-(butylene adipate-co-terephthalate) (PBAT) has become one of the most prevalent biodegradable plastic film materials owing to its good degradability, mechanical properties, and processability. However, the degradation time of this material was too fast and the functional period was short, which limited its application. Herein, three new tropolone-based UV absorbers (UVA-1C, UVA-4C and UVA-6C) were rationally designed and blended into PBAT. The PBAT/UVA films that formed were used against UV aging and prolonged the functional period of PBAT film. The three new absorbers were synthesized by bridging two tropolones using three different organic chains with different flexibility. Among them, the UVA-6C showed the strongest UV absorbance at around 238 nm and 320 nm. Consequently, the PBAT/UVA-6C film showed an extended validity period of 240 h in the Xenon lamp aging machine and a prolonged functional period of 8 d during the field application test when compared to pure PBAT. More importantly, a 7.8% increase in the maize yield was obtained under PBAT/UVA-6C film relative to pure PBAT film. Obviously, the novel prepared UVA-6C compound is a good candidate for UV absorption in PBAT, which makes PBAT/UVA-6C film more advantageous over pure PBAT in practical applications as biodegradable agricultural film.

## 1. Introduction

Plastic mulching film plays an important role in preserving moisture, increasing temperature, and controlling weeds in agricultural activities, and the demand for this agricultural material is huge and is steadily increasing. Normally, the conventionally used mulching film is polyethylene (PE) because of its easy process, low cost, good mechanical properties, etc. However, the leftover PE film is difficult to remove from the soil completely, so the widespread application of PE-type mulching films causes serious pollution to the environment [1,2]. Moreover, the high cost of recovery, in turn, decreased farmers’ incomes [3]. Therefore, a tremendous amount of research has been carried out to minimize such an environmental impact by replacing traditional mulching film with biodegradable ones [4,5]. Indeed, biodegradable polymers, such as polylactic acid (PLA) and Poly (butylene adipate-co-terephthalate) (PBAT), have been suggested for mulching film applications. Among them, Poly (butylene adipate-co-terephthalate) (PBAT) is more attractive because of its good biodegradability, mechanical properties, and processability [6]. In addition, PBAT is easy to process and has similar mechanical properties to that of PE [7]. Reasonably, it has been considered an ideal biodegradable agricultural film material [8,9]. However, PBAT is highly sensitive to photo-irradiation, which leads to photodegradation [10], while it easily decomposes approximately 30 days after it was used in the field [11]. The above disadvantages severely hinder the application of PBAT as an agricultural film [12]. Studies have shown that UV light causes the main damage to plastic films [13,14,15,16]. To minimize the negative effects of UV light, the use of UV absorbers as additives has been put forward to prolong the durability of plastic products and has obtained significant results [17,18]. Therefore, the use of UV absorbers as an additive in plastic is an effective strategy to enhance the UV aging resistance ability of plastics [19,20].

In the heat of the sun, a UV light absorber in plastic can transfer UV light to harmless energy such as heat after absorbing UV light, thus reducing the damage to films caused by UV rays. Presently, the existing UV absorbers in the market can be classified into inorganic UV and organic UV absorbers [21,22]. Regarding inorganic UV absorbers, it is not recommended to use them alone due to their low molecular weight and poor compatibility with the organic polymer matrix [23,24,25]. Organic UV absorbers mainly include, but are not limited to, small-molecule benzophenones, benzotriazoles, triazines, salicylates, etc. [26]. The ability of these UV absorbers to absorb UV rays is improved compared to inorganic UV absorbers. However, these organic UV absorbers show several defects, such as low molecular weight, resulting in easy migration from the polymer matrix during practical applications [27]. On one hand, the migration of UV absorbers from the matrix decreases the quality of plastic. On the other hand, the leached toxic small molecular absorber causes pollution to the environment. In addition, such kinds of low-molecular-weight UV absorbers were easy to decompose and/or volatilize during high-temperature polymer processing [28].

To solve these problems, there were reports of using inorganic nanoparticles to load organic UV absorbers and obtaining an obvious reduction in the migration rate. For example, a UV absorber (UV-P) was grafted onto carbon black (CB) by an in situ solid-state grafting method to obtain a modified UV absorber (UV-PCB), and a PETG/UV-PCB film was prepared with a stronger ability to absorb UV light than PETG/UV-P and PETG/CB films [29]. Similarly, S. Zhu et al. grafted 2, 4-dihydroxybenzophenone (UV-0) onto the surface of nano-silica (SiO_2_) and successfully prepared a new UV absorber (SiO_2_-N-0) [30]. The PBAT/SiO_2_-N-0 film showed stronger anti-aging ability compared with PBAT/UV-0, PBAT/SiO_2_ and PBAT/SiO_2_/UV-0 films. Nevertheless, the UV absorbers in the above studies did not consider extending the alkyl chains when designing the structures, although a longer alkyl chain may enhance its bonding with the polymer matrix. After decades of study, increasing the molecular weight of UV absorbers is an effective way to reduce their migration. However, most of the works involved tedious synthetic procedures. Herein, in this work, using three different organic carbon chains to connect two tropolone UV absorber centers, three novel UV absorbers (UVA-1C, UVA-4C and UVA-6C) with increased molecular weight were synthesized. The long organic carbon chains can make the combination of UV absorbers and polymers more stable and reduce the migration rate in the polymers. In addition, the larger molecular weight of UV absorbers will shield UV rays for a longer time. The novel UV absorbers were also used to prepare anti-UV-aging PBAT films, and a field application experiment of these films was conducted. Furthermore, the UV aging resistance, degradation behavior, stability, and mechanical strength of these films were investigated. This work provides a new way to enhance the UV aging resistance of biodegradable films, making them more suitable for field application.

## 2. Materials and Methods

### 2.1. Materials

PBAT (Mw: 93,227 g/mol; melting point: 127 °C) was purchased from Xinjiang Blue Ridge Tunhe Polyester Co., Ltd. (Xinjiang, China). Tropolone (C_7_H_6_O_2_), dibromo-p-xylene (C_8_H_8_Br_2_), potassium carbonate (K_2_CO_3_), DMF (N, N-dimethylformamide, C_3_H_7_NO), bisphenol A (C_15_H_16_O_2_), 1-bromo-4-chlorobutane (C_4_H_8_BrCl), 18-crown-6 (C_12_H_24_O_6_), 1-bromo-6-chlorohexane (C_6_H_12_BrCl), ethanol anhydrous (C_2_H_6_O), and sodium sulfate anhydrous (Na_2_SO_4_) were purchased from Shanghai Macklin Biochemical Technology Co., Ltd. (Shanghai, China). Ethyl acetate (C_4_H_8_O_2_), n-hexane (C_6_H_14_), and dichloromethane (CH_2_Cl_2_) were obtained from Da Mao Chemical Reagents Factory (Tianjin, China).

### 2.2. Preparation of UVA-1C, UVA-4C and UVA-6C

**Figure 1:**
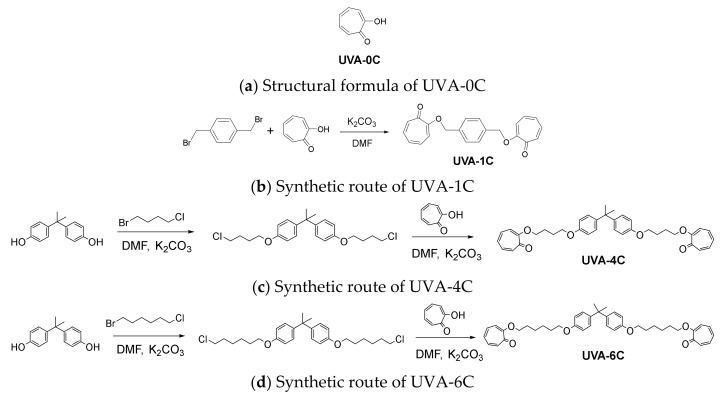
Synthetic routes of three UV absorbers.

The synthetic routes of UVA-1C, UVA-4C and UVA-6C are shown in Figure 1b–d, respectively.

**Synthesis of UVA-1C.** Tropolone (5.00 g, 41.00 mmol) and anhydrous potassium carbonate (6.50 g, 47.10 mmol) were dissolved in a three-necked flask containing 50 mL of anhydrous DMF and stirred at 25 °C for 30 min. Then dibromo-p-xylene (5.00 g, 18.90 mmol) was added to the above solution and the temperature was increased to 90 °C for 6 h. After cooling the above reaction solution to 25 °C, ice water was poured into the reaction mixture and fully stirred to quench the reaction. The obtained precipitation was the target product and was collected by filtration, washed with water, and dried in an oven. ^1^H NMR (400 MHz, CDCl_3_, δ ppm): δ 7.45 (s, 4H), 7.22–7.23 (m, 4H), 6.98 (t, J = 8.0 Hz, 2H), 6.85 (t, J = 12.0 Hz, 2H), 6.77 (d, J = 8.0 Hz, 2H), 5.25 (s, 4H). HRMS [M + Na] ^+^ calcd. for C_22_H_18_O_4_^+^ 369.1076; found: 369.1085. Anal. Cald. for C_22_H_18_O_4_: C, 76.29; H, 5.24; Found: C, 75.98; H, 5.13. ^1^H NMR and HRMS are shown in Figures S1 and S2.

**Synthesis of UVA-4C.** Reaction 1: Bisphenol A (5.00 g, 21.90 mmol) and anhydrous potassium carbonate (18.30 g, 132.60 mmol) were dissolved in a three-necked flask containing 50 mL anhydrous DMF and stirred at 25 °C for 30 min. Then, 1-bromo-4-chlorobutane (9.40 g, 55.00 mmol) was dropped into the three-necked flask and the temperature was increased to 70 °C for 9 h to obtain the intermediate. After cooling the above reaction solution to 25 °C, ice water was poured into the reaction mixture and fully stirred to quench the reaction and was then extracted with ethyl acetate. After liquid separation, the organic phase was dried with sodium sulfate anhydrous and concentrated to obtain the crude intermediate of the first step reaction. Purification: The crude product obtained was purified by silica gel column chromatography. The solvents used in this operation were n-hexane and ethyl acetate (80:1–5:1), and the target product of Reaction 1 was obtained. Reaction 2: Cycloheptene phenol ketone (4.48 g, 36.75 mmol) and anhydrous potassium carbonate (5.05 g, 36.59 mmol) were dissolved in anhydrous DMF (50 mL) and 18-crown-6 (0.50 g, 1.89 mmol) in a three-necked flask, and the mixture was stirred for 30 min at 25 °C. Then, the pure intermediate (7.14 g, 15.40 mmol) obtained in Reaction 1 was dropped into the three-necked flask and the temperature was increased to 90 °C for 9 h. The subsequent cooling and purification operation steps were the same as those in Reaction 1. Finally, a brown oily target product was obtained. ^1^H NMR (400 MHz, CDCl_3_, ppm): δ 7.20 (d, J = 8.0 Hz, 4H), 7.12 (d, J = 8.0 Hz, 4H), 7.03 (t, J = 8.0 Hz, 2H), 6.86–6.81 (m, 2H), 6.78 (d, J = 8.0 Hz, 4H), 6.73 (d, J = 12.0 Hz, 2H), 4.13 (t, J = 8.0 Hz, 4H), 4.01 (t, J = 8.0 Hz, 4H), 2.14–2.07 (m, 4H), 2.01–1.95 (m, 4H), 1.63 (s, 6H). HRMS [M + Na] ^+^ calcd. for C_37_H_40_O_6_^+^ 603.2722; found: 603.2716. Anal. Cald. for C_37_H_40_O_6_: C, 76.53; H, 6.94; Found: C, 76.38; H, 6.87. ^1^H NMR and HRMS are shown in Figures S1 and S2.

**Synthesis of UVA-6C.** The synthetic route of UVA-6C was very similar to that of UVA-4C. Therefore, it will not be repeated. The final pure target product was also a brown oily pure product. ^1^H NMR (400 MHz, CDCl_3_, ppm): δ 7.20 (d, J = 4.0 Hz, 4H), 7.12 (d, J = 8.0 Hz, 4H), 7.03 (t, J = 12.0 Hz, 2H), 6.85–6.80 (m, 2H), 6.78 (d, J = 8.0 Hz, 4H), 6.74 (s, 1H), 6.71 (s, 1H), 4.05 (t, J = 8.0 Hz, 4H), 3.93 (t, J = 8.0 Hz, 4H) 1.98–1.91 (m, 4H), 1.82–1.76 (m, 4H), 1.63 (s, 6H), 1.56–1.52 (m, 8H). HRMS [M + Na] ^+^ calcd. for C_41_H_48_O_6_^+^ 659.3330; found: 659.3343. Anal. Cald. for C_41_H_48_O_6_: C, 77.33; H, 7.60; Found: C, 77.18; H, 7.53. ^1^H NMR and HRMS are shown in Figures S1 and S2.

Furthermore, it is worth mentioning that UVA-0C, shown in Figure 1a, was a commercially available product called tropolone (appeared in Section 2.1).

### 2.3. Preparation of PBAT/UVA Composite Films

The series of PBAT/UVA films were prepared by blow molding, and their compositions are shown in Table 1. Firstly, PBAT pellets were dried at 80 °C for 4 h before use. Taking PBAT/UVA-0C as the representative example, physical mixing of the UVA-0C with PBAT followed by squeezing and pelletizing using a double-screw extruder (LTE-26-44, Labtech Engineering, Praksa, Thailand) and a pelletizer (LZ-120/vs, Labtech Engineering, Praksa, Thailand), respectively, were carried out, obtaining a PBAT/UVA-0C masterbatch. The pelletized temperature was approximately 150 °C. After that, the obtained masterbatches were loaded onto the blown film machine (LF400-COEX, Labtech Engineering, Praksa, Thailand) to produce PBAT/UVA-0C films, whereby the blowing temperature was approximately 160 °C from zone 1 to zone 5, and the thickness of all films was 10 ± 1 μm. PBAT/UVA-1C, PBAT/UVA-4C and PBAT/UVA-6C were prepared in the same way, and the pure PBAT film was selected as the reference sample.

### 2.4. Characterization and Property Tests

#### 2.4.1. HRMS

HRMS (High-Resolution Mass Spectrometry) was used to separate the compounds by a Triple Quadrupole MS (Waters India Pvt Ltd., Karnataka, India). The mobile phase consisted of 0.1% formic acid in water with acetonitrile as the co-solvent. MS was used in both positive and negative polarity using an ESI source with a mass range of 80–1000 Daltons.

#### 2.4.2. ^1^H NMR

^1^H NMR was determined by the Advance Bruker III 400 (400 MHz) NMR spectrometer, tetramethylsilane (TMS) was used as the internal standard, and CDCl_3_ was used as the solvent.

#### 2.4.3. Carbon Hydrogen Elements Analysis

The carbon hydrogen elements of newly synthesized UV absorbers were analyzed by a carbon hydrogen elements analyzer (Vario EL cube, Elementar, Germany).

#### 2.4.4. FTIR Analysis

Fourier transform infrared (FTIR) spectroscopy was collected on a Nicolet IS10 FTIR (Thermo Fisher, MA, USA) infrared spectrometer in the 400–4000 cm^−1^ range using the KBr disk method.

#### 2.4.5. UV-Vis Spectral Analysis

UV–vis spectroscopy was performed on a UV-3600 UV (Shimadzu EMIT Co., Ltd., Tokyo, Japan) spectrometer in the 200–800 nm range. The solvent used during the UVA samples analysis was dichloromethane.

#### 2.4.6. TGA/DTG Analysis

The thermogravimetric analysis (TGA) and derivative thermogravimetry (DTG) analysis were determined by a thermogravimetric analyzer (sta449f3, Netzsch, Bavarian Asia, Germany) in the 25 to 800 °C range with a heating rate of 10 °C/min under a nitrogen atmosphere.

#### 2.4.7. AFM Analysis

The test was analyzed by an atomic force microscope (AFM) (Dimension Icon, Bruker, Germany) to analyze the interatomic force and surface roughness of films. It was operated in tapping mode, the scanning frequency was 1 Hz, the scanning area was 5 μm × 5 μm, and the test temperature was 25 °C.

#### 2.4.8. Weathering Test

According to ASTM G155-2013, the accelerated photoaging test was carried out on a xenon weatherometer (BGD866, Biuged laboratory instruments Co., Ltd., Guangzhou, China), and the program setting was executed according to Cycle 1. The cumulative duration of the experiment was 600 h, and film samples were taken out every 120 h for visual analysis, taking photos, gel permeation chromatography, FTIR, and UV-Vis analysis.

#### 2.4.9. Visual Analysis

The visual analysis was performed by visual observation and photographic recording. The film samples in the xenon weatherometer needed to be taken out regularly (every 120 h) and photographed to record the degradation of the films.

#### 2.4.10. GPC Analysis

Gel permeation chromatography (GPC) was determined by the molecular weight of film samples at different degradation stages. The solvent was tetrahydrofuran (THF), the mobile phase flow was 1 mL/min, the temperature was 40 °C, and the injection volume was 100 μL. The standard sample was narrow-distribution polystyrene (PS) (TOSOH, Yamaguchi, Japan).

#### 2.4.11. Carbonyl Index Analysis

The carbonyl index (CI) was used to evaluate the degradation degree of PBAT films. CI was collected on the Bruker OPUS 7.5 spectrometer (Bruker Instruments Co., Ltd., Karlsruhe, Germany) in the 4000 to 600 cm^−1^ range and the mode was ATR. CI was calculated as follows:

(1) $$\mathrm{CI}=\frac{\mathrm{Ac}}{\mathrm{Ar}}$$

Ac was the absorbance of the carbonyl vibration peak at 1715 cm^−1^ and Ar was the absorbance of multiple -CH_2_- groups at 729 cm^−1^.

#### 2.4.12. The Migration Rate of UVA in Films

The migration rate of the PBAT/UVA films was measured by recording the UV absorption spectrum of the film sample soaked in ethanol solution. In detail, four pieces of film of the same size were taken from PBAT/UVA-0C, PBAT/UVA-1C, PBAT/UVA-4C and PBAT/UVA-6C, respectively. Then, the four pieces of films were soaked in four different beakers, each containing 30 mL of absolute ethanol. Each film sample was soaked for 30 min, 60 min, 120 min, 180 min and 300 min; 5 mL of the soaked solution was sampled at each time point, and the UV-3600 was used to record the UV absorption spectrum of the soaked solution. The migration rate of each sample was determined by calculating the absorbance intensity of the released UV absorber.

#### 2.4.13. Mechanical Properties

The mechanical properties of PBAT films were recorded on a universal testing machine (Labthink Co., Ltd., Jinan, China). The tensile and tear properties test met the requirements of ASTM D882-2018 and ASTM D1004-2013, respectively. Moreover, the film thickness test met the ASTM D6988-2013 standard and was carried out on a Millimar C1200 Thickness Gauge (Mahr Ltd., Shanghai, China). In all the above tests, each film sample was tested 5 times.

#### 2.4.14. Water Vapor Transmission Rate

The water vapor transmission rate was collected on a W3/060 PERME machine (Labthink, Jinan, China). All films samples were cut to 33.2 cm^2^ and placed in a test chamber under a temperature of 38 °C and humidity of 90% according to ASTM E96/E96M-2014. Each film sample was tested twice.

### 2.5. Field Application Evaluation of PBAT/UVA Films

#### 2.5.1. Field Experiment Design

The experiment was conducted in the Shunyi experimental station of the Chinese Academy of Agricultural Sciences, Beijing, China (40°09′ N, 116°52′ E). The site had a warm temperate and semi-humid monsoon climate, with an annual average temperature of 12 °C, annual sunshine of 2750 h, and an annual number of frost-free days of 195. The station had an annual average rainfall of 625 mm, mainly from June to August. The experimental site soil was meadow cinnamon soil with 64.0% silt, 29.0% sand and 7.0% clay, and was mainly used to produce wheat (*Triticum aestivum* L.) and maize (*Zea mays* L.). The physical and chemical properties of soil (0–30 cm depth) was checked before sowing, and the soil contained total organic carbon of 14.0 g/kg, total nitrogen of 0.9 g/kg, available nitrogen of 158.0 mg/kg, bulk density of 1.20 g/cm^3^, and a pH value of 7.9.

A total of seven treatments were tested, including CK (no film), PE film, PBAT film, PBAT/UVA-0C film, PBAT/UVA-1C film, PBAT/UVA-4C film and PBAT/UVA-6C film, with each test for each sample being repeated three times. The tested crop was fresh maize (NONGKENUO 336, 20200021), and the planting density was approximately 3000 stems/667 m^2^. The planting method of double ridges on films was adopted. The spacing between the two ridges on films was 45 cm and the spacing between two maize plants was 25 cm. The total area of the test site was approximately 1135 m^2^, and the test area of each kind of film was approximately 160 m^2^ (three groups of 50-m-long films were laid). All field management measures were consistent, and the only variable in this experiment was film samples. The experimental period lasted from 25 April 2021 to 22 July 2021.

#### 2.5.2. The Functional Period of Films

Three observation points were fixed on each film sample, with an area of 50 cm × 50 cm. From the beginning of film covering, we observed the changes in the films’ surface every week (holes, cracks and breakage) and recorded photos with a digital camera (fixed height of 50 cm overhead). Once the broken area of more than 20 cm accounted for 30% of the whole field film area and resulted in the exposure of the soil, the functional period of the film was determined.

#### 2.5.3. Soil Temperature under Films

An automatic temperature recorder (HOBO U22-001, Onset Co., Wareham, MA, USA) was used for soil temperature measurements. One recorder was embedded in each plot (embedded during sowing). The buried depth of the probe was approximately 10 cm. The recorder recorded the data every 30 min. After maize was harvested, we dug out the recorder and exported the data for further use.

#### 2.5.4. Maize Yield under Films

After the maize was mature, 5 m was selected randomly from each plot and we counted the number of corn plants, harvested all corn, and weighed them (fresh weight). Then, the net weight of corn was converted into hectare yield in the unit of kg/hm^2^, accurate to 0.1 kg/hm^2^. Each plot was repeated three times.

## 3. Results and Discussion

### 3.1. Characterization of UVA

#### 3.1.1. FTIR Analysis of UVAs

**Figure 2:**
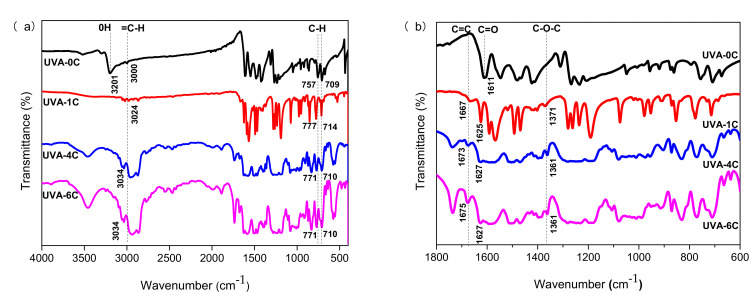
FTIR spectrum of UVAs ((**a**) 4000–400 cm^−1^; (**b**) 1800–600 cm^−1^).

Figure 2 shows the FTIR spectra of UVA-0C, UVA-1C, UVA-4C and UVA-6C, which were mainly used to analyze the main functional groups in UV absorbers. The main functional peaks in UVA-0C were an -OH vibration peak at 3201 cm^−1^, a C-H vibration peak at 3000 cm^−1^, and a -C=O vibration peak at 1611 cm^−1^. The main peaks in UVA-1C, UVA-4C and UVA-6C were a =C-H vibration peak near 3024 cm^−1^, a -C=C- peak near 1667 cm^−1^, a -C=O peak near 1625 cm^−1^, and a -C-O-C- peak near 1371 cm^−1^. Besides, the UVA-4C and UVA-6C showed a broad peak around 3500 cm^−1^, belonging to the phenolic [21]. The vibrational peaks of these characteristic functional groups showed that the structures of the newly prepared UV absorbers were reasonable. In summary, the comprehensive results of HRMS, ^1^H NMR, carbon hydrogen elements analysis, and FTIR proved that the molecular structure of the newly synthesized UV absorbers was correct and the UV absorbers were successfully prepared.

#### 3.1.2. UV-Vis Spectral Analysis of UVAs

**Figure 3:**
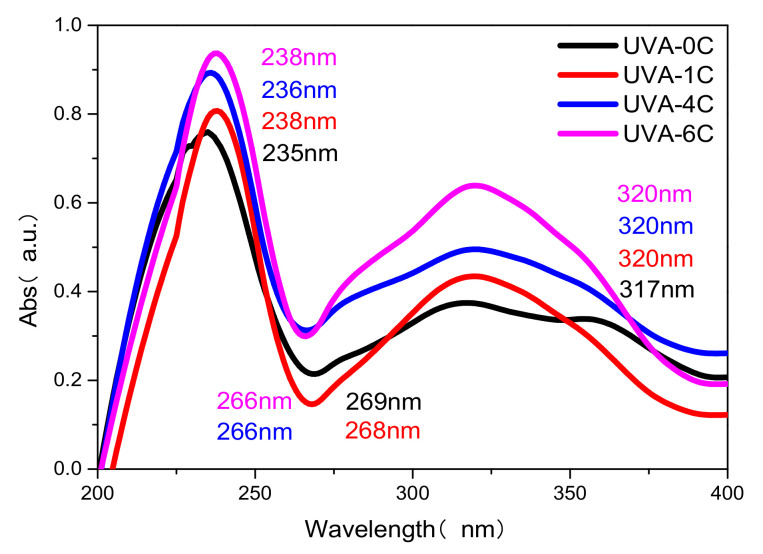
UV-Vis spectral of UVAs.

The UV absorption spectra of UVA-0C, UVA-1C, UVA-4C and UVA-6C are shown in Figure 3. The number of moles of UV absorption centers in each compound solution is the same during the test. As shown in Figure 3, the four UV absorbers have similar maximum absorption peaks at approximately 238 nm and 320 nm, and also have two similar UV absorption bands from 210 to 260 nm and from 270 to 370 nm, corresponding to n-π* transitions [31]. The results indicated that the four UV absorbers can effectively absorb multi-band UV light and possess a satisfactory UV absorption capacity. To be noted, the maximum absorption peaks of the organic carbon chain connected UV absorbers slightly redshift to a longer wavelength when compared with UVA-0C, and this change may be due to the substituents that play a role in electron donating. In addition, it is not hard to find that the order of UV absorption intensity was UVA-6C > UVA-4C > UVA-1C > UVA-0C. These differences can be explained by the molecular weight and chromogenic groups (-C=O- and -C=C-). The UVA-6C had the largest molecular weight and contained more chromogenic groups in its structure compared with the other three. Therefore, its UV absorbance value was the highest.

#### 3.1.3. TG/DTG Analysis of UVAs

**Figure 4:**
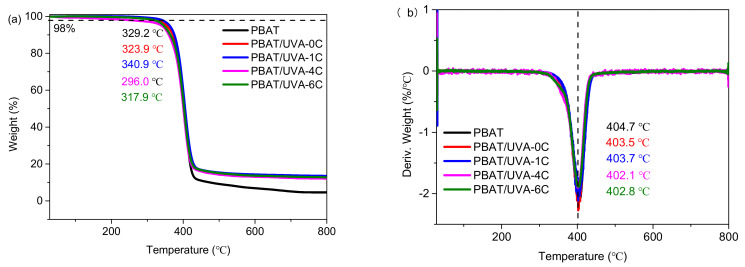
TG (**a**) and DTG (**b**) curves of UVAs.

To investigate the thermal stability of absorbers UVA-0C, UVA-1C, UVA-4C and UVA-6C, thermogravimetric (TG) and derivative thermogravimetry (DTG) analyses were carried out. As displayed in Figure 4a, UVA-0C, UVA-1C, UVA-4C and UVA-6C have an onset decomposition temperature of 87.7 °C, 167.0 °C, 285.4 °C and 290.2 °C, respectively. These results indicate that the introduction of an organic carbon chain between two tropolones can improve the thermal stability of the corresponding compounds, especially for the UVA-4C and UVA-6C absorbers, both of which have high decomposition temperatures that are stable enough for further film fabrication. Compared with UVA-0C, the improved thermal stability of new types of absorbers may come from the greater molecular weight and the substitution effect in which organic carbon chains substitute the hydrogen atom on the hydroxyl group, which leads to a more stable structure of tropolone. Figure 4b shows the DTG properties of the four absorbers. As is depicted, UVA-0C and UVA-1C reached a maximum decomposition rate at relatively low temperatures of 177.5 °C and 230.5 °C, respectively. Regarding the UVA-4C and UVA-6C compounds, both of them have two different maximum decomposition rates of 337.6 °C and 417.4 °C, 339.2 °C and 434.2 °C, respectively. This indicated that UVA-4C and UVA-6C possess multistep decomposition. Specifically, temperatures of 337.6 °C and 339.2 °C likely corresponded to the fracture decomposition of alkyl chains in UVA-4C and UVA-6C, respectively. Considering that there was residual carbon in the last decomposition step, the weight loss at 417.4 °C and 434.2 °C may come from the release of CO and CO_2_ [32]. As is mentioned in the characterization part, the blow-molding temperature for the PBAT film was approximately 150 °C, so the UV absorbers will not be decomposed during film fabrication. So, it is safe to mix UV absorbers with PBAT to prepare films.

### 3.2. Characterization of PBAT and PBAT/UVA Films

#### 3.2.1. FTIR Analysis of Films

**Figure 5:**
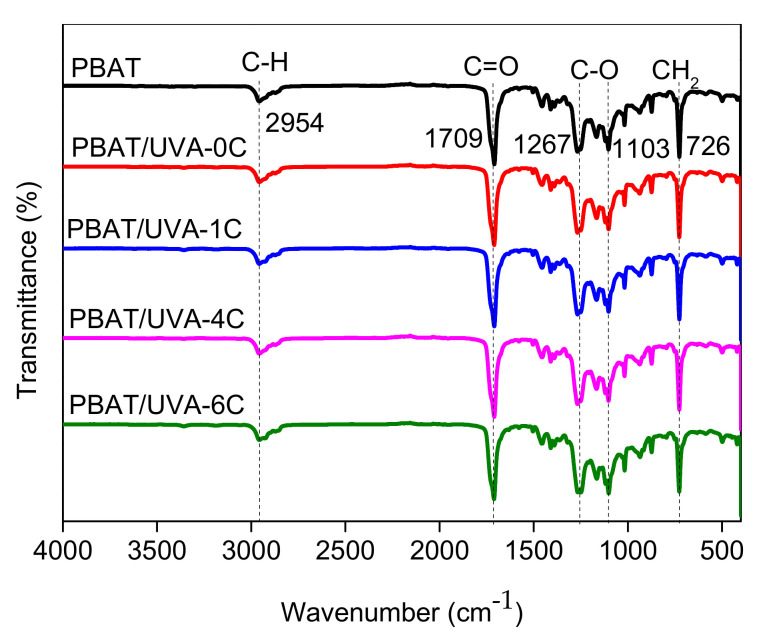
FTIR spectra of PBAT and PBAT/UVA films.

The PBAT and the newly prepared PBAT/UVA film were investigated by FTIR. As shown in Figure 5, the absorption peak at 2954 cm^−1^ of PBAT was assigned to the stretching vibration of -CH_2_-, and the absorption peak at 1715 cm^−1^ was attributed to the stretching vibration of C=O. The peaks at 1268 cm^−1^ and 1103 cm^−1^ correspond to the stretching vibration of C-O-C. In addition, the sharp absorption peak at 726 cm^−1^ was the absorption peak of four or more adjacent -CH_2_- groups. These characteristic peaks were consistent with Q. Zhang’s study [33]. For the PBAT/UVA-0C, PBAT/UVA-1C, PBAT/UVA-4C and PBAT/UVA-6C films, their spectrum was almost the same as PBAT, and there were no addition of new functional groups and no reduction of the original groups. These results revealed that the loading amount of UV absorbers in the film is too low to detect.

#### 3.2.2. Atomic Force Microscope Analysis (AFM)

**Figure 6:**
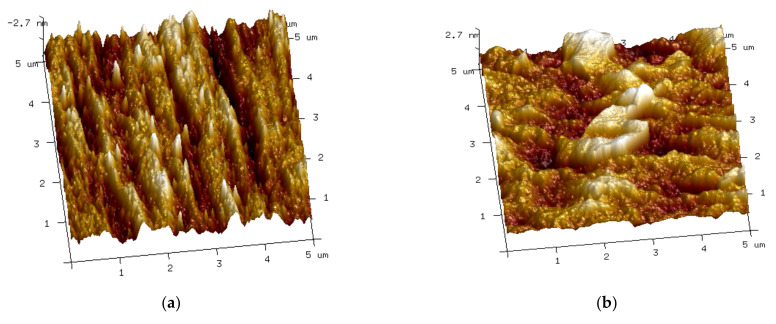

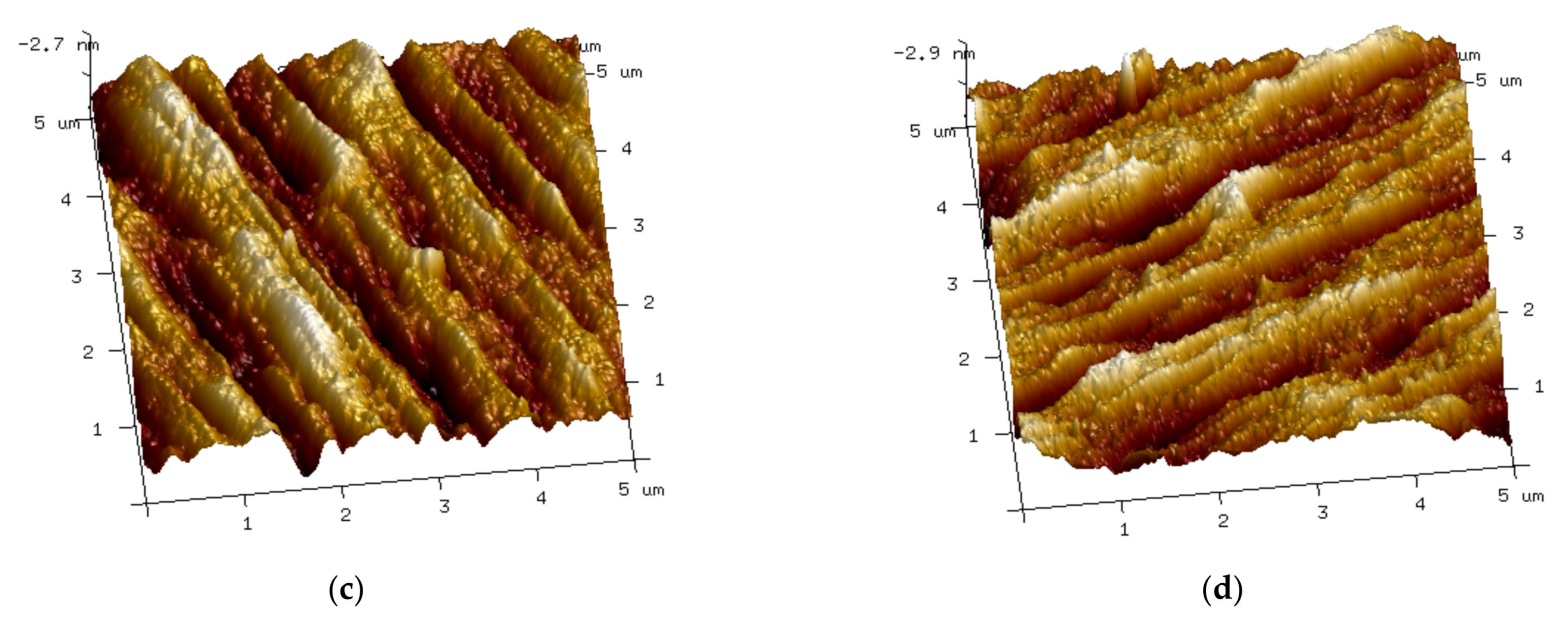
AFM images of PBAT/UVA films’ surface, ((**a**) PBAT/UVA-0C, (**b**) PBAT/UVA-1C, (**c**) PBAT/UVA-4C, (**d**) PBAT/UVA-6C).

To study the dispersion of UVAs in the PBAT matrix and the force between PBAT and UVAs, the AFM images were scanned and are shown in Figure 6. The values of surface roughness were tested in the same height range (−20.0 nm–20.0 nm). From 6a–d, obvious gullies and sharp protrusions can be seen on the surface of the PBAT/UVA-0C film, while the surfaces of PBAT/UVA-1C, PBAT/UVA-4C and PBAT/UVA-6C films became flatter and more evenly distributed. The values of surface roughness of PBAT/UVA-0C, PBAT/UVA-1C, PBAT/UVA-4C and PBAT/UVA-6C films were 11.9 nm, 11.5 nm, 10.8 nm and 9.5 nm, respectively. The small value of roughness represented the flatness and good dispersion of the film surface. The experimental results illustrated that the distribution of UVA-6C in PBAT film was the most uniform compared with other films. In other words, the atomic force between UVA-6C and the PBAT matrix was relatively strong, and the combination between the two was the closest. The migration and precipitation rate of UVA-6C from PBAT was the slowest in the same batch. This was consistent with the assumptions made when designing the UVA-6C structure.

#### 3.2.3. Visual Analysis of Films in Xenon Weatherometer

The photos in Table 2 present the degradation degree of PBAT and PBAT/UVA films at different aging times. As listed in Table 3, the pure PBAT sample reached grade 1 after 240 h. At 360 h, the degradation degree of PBAT reached grade 2, PBAT/UVA-0C and PBAT/UVA-1C reached grade 1, while PBAT/UVA-4C and PBAT/UVA-6C were still at grade 0. When the aging time was prolonged to 480 h, the conditions of PBAT, PBAT/UVA-0C, PBAT/UVA-1C, PBAT/UVA-4C and PBAT/UVA-6C progressed to grade 3, grade 2, grade 2, grade 1 and grade 1, respectively. After aging for 600 h, the degradation degree of the pure PBAT film was reached grade 4, where the integrity of the film was broken and cracked into pieces, while the other four film samples were still in good condition. In particular, PBAT/UVA-4C and PBAT/UVA-6C had no large area fracture. These results showed that the degradation rate of pure PBAT was the fastest, and the existence of UVA in the PBAT/UVA films perceptibly reduced the degradation rate.

During the degradation evolution of these films, we found that the ability to increase temperature and preserve the moisture of soil was lost to a certain extent when the degradation degree of films reached grade 2. Therefore, grade 2 was defined as the validity period of film function. Based on this, it can be concluded that the validity period of PBAT/UVA-0C and PBAT/UVA-1C was 120 h longer than PBAT, and the validity period of PBAT/UVA-4C and PBAT/UVA-6C was 240 h longer than PBAT. As mentioned above, the existence of UV absorbers greatly enhanced the anti-UV-aging ability of PBAT films.

#### 3.2.4. UV-Vis Absorption Spectrum of Films

**Figure 7:**
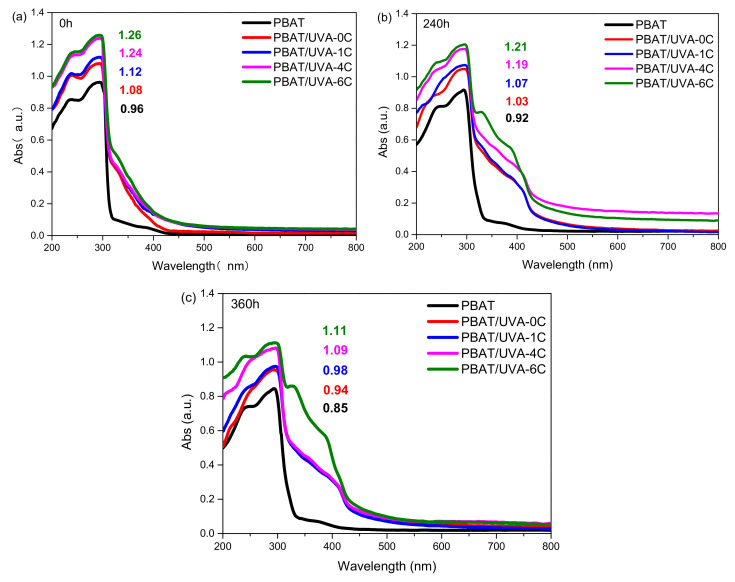
UV-vis spectra of PBAT and PBAT/UVA films. ((**a**): Aging for 0 h; (**b**): Aging for 240 h; (**c**): Aging for 360 h).

Figure 7 presents the UV–vis spectra of PBAT and PBAT/UVA films. In the wavelength range of 400–800 nm, the absorbance of all films was less than 0.2, which indicated that the introduction of UV absorbers had little effect on the light transmission performance of PBAT films. From Figure 7a, the absorption peaks of PBAT films were mainly located between 200 and 400 nm in the UV region [35], and the absorbance of the PBAT, PBAT/UVA-0C, PBAT/UVA-1C, PBAT/UVA-4C and PBAT/UVA-6C films were 0.96, 1.08, 1.12, 1.24 and 1.26, respectively. Moreover, the PBAT/UVA-0C, PBAT/UVA-1C, PBAT/UVA-4C and PBAT/UVA-6C films increased by 11.1%, 14.3%, 22.6% and 23.8%, respectively, compared to that of PBAT. This implied that the films with added UVA were able to absorb UV and employ an anti-aging effect more effectively.

After UV aging for 240 h and 360 h, the UV absorption intensity of all films decreased. The PBAT, PBAT/UVA-0C, PBAT/UVA-1C, PBAT/UVA-4C and PBAT/UVA-6C decreased by 4.2%, 4.6%, 4.5%, 4.0% and 4.0% after 240 h and decreased by 12.9%, 14.9%, 14.3%, 13.8% and 13.5% after 360 h, respectively. This was due to the polymer decomposing by UV light after UV aging, which was consistent with Wang Yang’s research [36]. From the molecular level, it was caused by the fracture of the organic carbon chains and the reduction in chromogenic groups. Because UVA-6C (the absorber that worked best among the tested batch) combined more closely with PBAT, it was able to effectively shield UV light and played a role for the longest time. As for PBAT, the number of chromogenic groups was less, and the degree of decline was limited. Perhaps this was the reason for the low degree of decrease in the decline rate. Therefore, the sort of degradation rate and service cycle comprehensive performance of all films decreased as follows: PBAT/UVA-6C > PBAT/UVA-4C > PBAT/UVA-1C > PBAT/UVA-0C > PBAT.

#### 3.2.5. GPC of Films

Table 4 listed the changes in molecular weight before and after PBAT and PBAT/UVA film aging. The change level of molecular weight reflected the degradation degree of films. Due to the serious degradation of the samples and very little residual left, the GPC test was not carried out after aging for 600 h. According to the information in Table 4, the Mn of each film decreased gradually with the extension of aging time. These results showed that the films had been degraded, the long chain broke, and the number of substances with low molecular weight increased with the increase in time [37,38]. Besides, the decline degree of Mn was also given in Table 4. At the same aging time, the decline rate of PBAT was always higher than that of the other four films. It illustrated that adding UVA into the film could greatly reduce the change in molecular weight and effectively slow down the degradation rate of films. The maximum decline degree of Mn of PBAT and PBAT/UVA-0C occurred at 240 h, reaching 66.7% and 54.9%, respectively. Then, the maximum degradation degree of PBAT/UVA-1C, PBAT/UVA-4C and PBAT/UVA-6C occurred between 240 h and 360 h and decreased by 45.6%, 35.0% and 29.3%, respectively. At 480 h, the decline degree of Mn of PBAT reached 91.3%, which had almost degraded completely, while for the other four PBAT/UVA films, the decline degrees were all more than 60%. This reflected that PBAT/UVA-1C, PBAT/UVA-4C and PBAT/UVA-6C have a stronger anti-aging ability than PBAT and PBAT/UVA-0C. Therefore, the anti-aging ability of these films decreased as follows: PBAT/UVA-6C > PBAT/UVA-4C > PBAT/UVA-1C > PBAT/UVA-0C > PBAT. This was consistent with the analysis results in the above visual analysis and UV-Vis analysis.

#### 3.2.6. Carbonyl Index (CI) of Films

**Figure 8:**
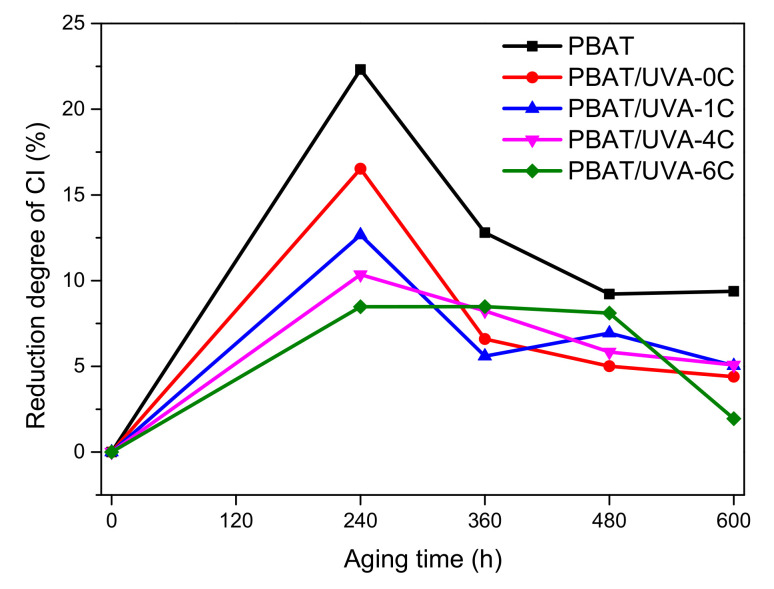
Decreasing degree curves of CI of PBAT and PBAT/UVA films.

The decreasing degree curves of the carbonyl index of PBAT and PBAT/UVA films are shown in Figure 8. CI expressed the absorbance of carbonyl relative to the reference band, and its decrease indicated the formation and migration of low-molecular-weight components [39]. Y. Nie’s research mentioned that the carbonyl index could be used as an index to evaluate the degradation degree of plastic films [40]. From the curve trend in Figure 8, after aging for 240 h, the CI of PBAT, PBAT/UVA-0C, PBAT/UVA-1C, PBAT/UVA-4C and PBAT/UVA-6C films decreased the most. CI decreased by 22.3%, 16.5%, 12.7%, 10.4% and 8.5%, respectively. This could be attributed to the maximum chain scission during aging. For durations of 360 h, 480 h and 600 h, the CI decline degree of all films slowed down when compared with 240 h. The possible reason was that there were many sites that can be attacked in the first period of aging time. After that, the available reaction sites were saturated, so the decline rate became low. Throughout the whole aging period, the CI decreased in the following orders pure PBAT > PBAT/UVA-0C > PBAT/UVA-1C > PBAT/UVA-4C > PBAT/UVA-6C, while the degradation degree of films was the same from large to small. These results are in accordance with the conclusions obtained in UV-Vis and GPC above. The introduction of UV absorbers in PBAT could effectively reduce the decline degree of CI, in other words, decreasing the degradation degree of films.

#### 3.2.7. The Migration of UV Absorbers in Films

**Figure 9:**
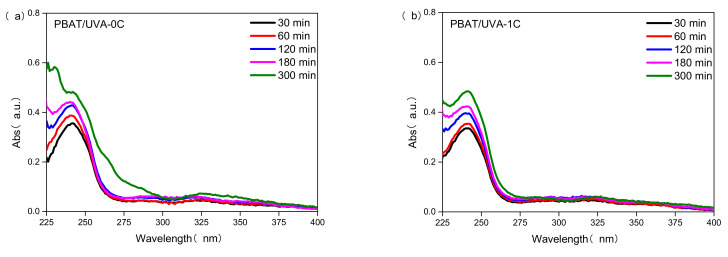

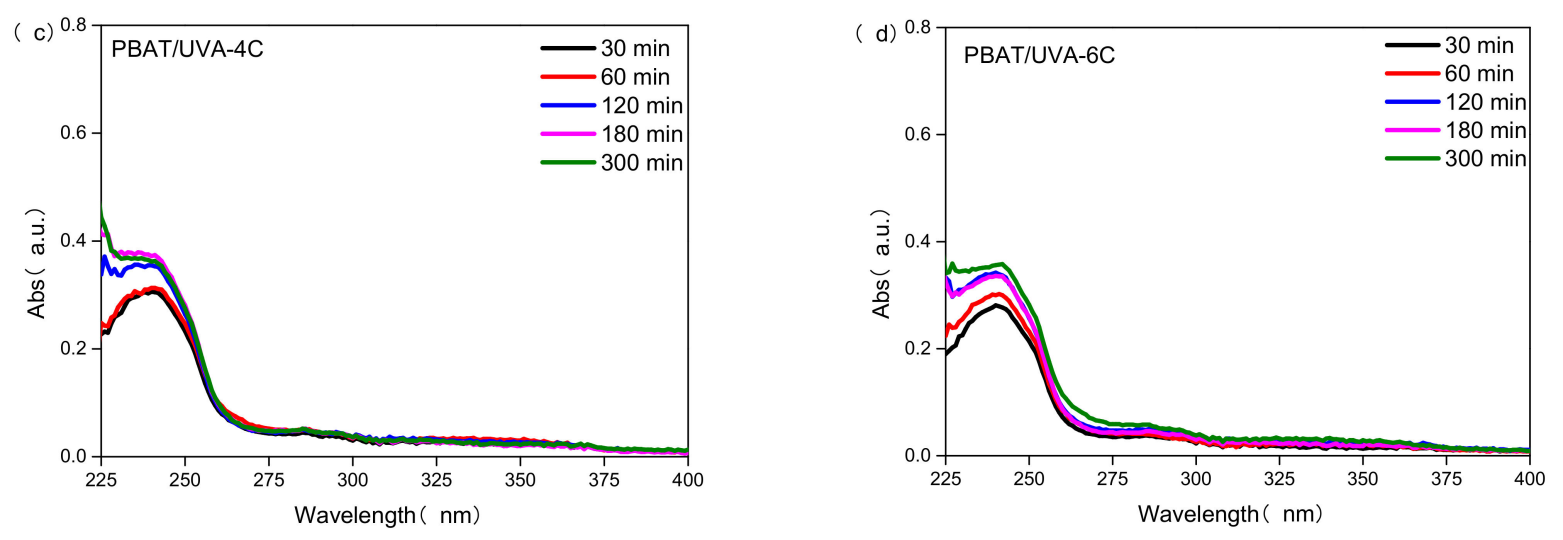
The migration rate curves of UVA in PBAT/UVA films at different times. ((**a**): PBAT/UVA-0C film; (**b**): PBAT/UVA-1C film; (**c**): PBAT/UVA-4C film; (**d**): PBAT/UVA-6C film).

To study the stability of UV absorbers in films, the UV absorption of the ethanol-soaked PBAT/UVA-0C, PBAT/UVA-1C, PBAT/UVA-4C and PBAT/UVA-6C films at different times are shown in Figure 9. The higher the absorbance, the faster the migration and precipitation rate of UV absorbers, and the worse the stability of UVAs in films. The UV absorption intensity of four films all increased over time, indicating that the UV absorbers in the four films migrated and larger amounts of migration accumulated. At 300 min, the UV absorption intensities of PBAT/UVA-0C, PBAT/UVA-1C, PBAT/UVA-4C and PBAT/UVA-6C films were 0.6, 0.5, 0.38 and 0.36, respectively, which indicated that the UVA in the PBAT/UVA-0C film migrated the most and its stability in the film was the worst. The most-stable film was PBAT/UVA-6C, followed by PBAT/UVA-4C and PBAT/UVA-1C, which were 40.0%, 36.7% and 16.7% lower than PBAT/UVA-0C, respectively. The good stability of the UVA-6C in PBAT/UVA-6C film may relate to the special structure of UVA. The structural formula of UVA-6C contained longer alkyl chains with good flexibility and strong fluidity, which brought it into closer contact with PBAT. In other words, when there was an external force, for example, UV light, the UVA-6C and PBAT were not easily separated, so the stability was enhanced.

#### 3.2.8. Mechanical Properties of Films

**Figure 10:**
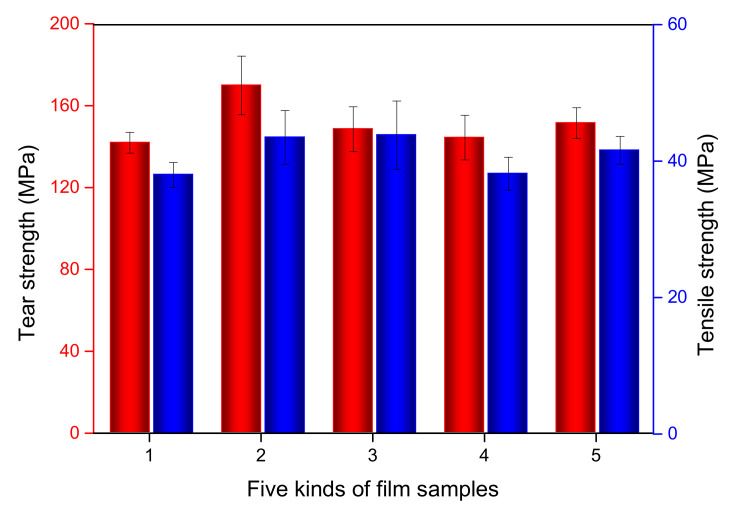
Mechanical properties of PBAT and PBAT/UVA films. (Red column: tear strength; Blue column: tensile strength; 1: PBAT, 2: PBAT/UVA-0C, 3: PBAT/UVA-1C, 4: PBAT/UVA-4C, 5: PBAT/UVA-6C).

The tensile strength and tear strength properties of PBAT and freshly prepared PBAT/UVA serial films were tested. As is displayed in Figure 10, the tensile strength of PBAT, PBAT/UVA-0C, PBAT/UVA-1C, PBAT/UVA-4C and PBAT/UVA-6C was 38.0 MPa, 43.5 MPa, 43.8 MPa, 38.1 MPa and 41.6 MPa, respectively. For the tear strength, PBAT, PBAT/UVA-0C, PBAT/UVA-1C, PBAT/UVA-4C, and PBAT/UVA-6C exhibit a value of 141.9 KN/m, 169.9 KN/m, 148.5 KN/m, 144.4 KN/m and 151.5 KN/m, respectively. The results indicated that the tensile strength and right-angle tear strength of all films met the field application requirements of mulching films. In addition, the addition of UVAs had little effect on the mechanical properties of plastic films.

#### 3.2.9. Water Vapor Transmission of Films

**Figure 11:**
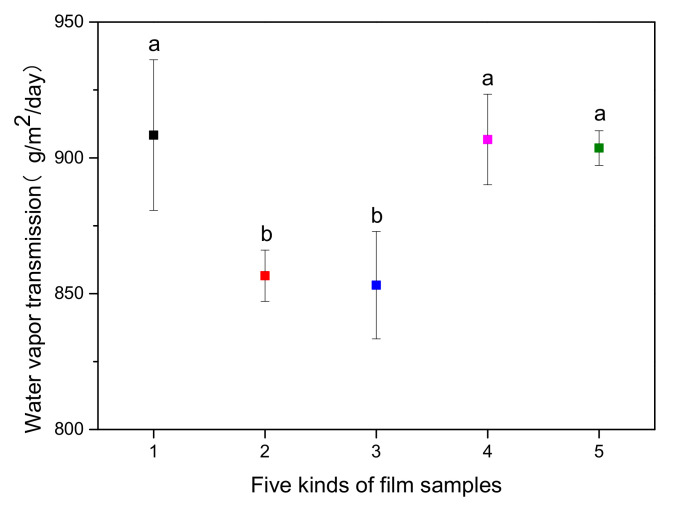
Water vapor transmission of PBAT and PBAT/UVA films. (1: PBAT, 2: PBAT/UVA-0C, 3: PBAT/UVA-1C, 4: PBAT/UVA-4C, 5: PBAT/UVA-6C; the lower-case letter in the figure shows a significant difference at the level of *p* < 0.05 (Duncan’s new multiple range test)).

To investigate the water retention performance of films, the water vapor transmission properties of the serials of PBAT films before aging are shown in Figure 11. The low water vapor transmittance of films indicated that they had excellent water retention performance. The values in PBAT, PBAT/UVA-0C, PBAT/UVA-1C, PBAT/UVA-4C and PBAT/UVA-6C were 908.4, 856.6, 853.2, 906.8 m and 903.6 g/m^2^·24 h, respectively. Obviously, the water vapor transmittance of films with UV absorbers was lower than that of PBAT, and there was a 5.7% decrease in PBAT/UVA-0C, a 6.1% decrease in PBAT/UVA-1C, a 0.2% decrease in PBAT/UVA-4C, and a 0.5% decrease in PBAT/UVA-6C. This meant that the introduction of UV absorbers was able to reduce the water vapor transmittance of PBAT. Compared with PBAT/UVA-4C and PBAT/UVA-6C, PBAT/UVA-0C and PBAT/UVA-1C were more significant regarding the reduction of water vapor transmittance. This discrepancy could be attributed to the properties of UV absorbers. UVA-0C and UVA-1C were fine, solid particles and had a low addition concentration, which could better fill the voids in the PBAT matrix and reduced the porosity of the film’s surface. Therefore, the effect of reducing the water vapor transmission was achieved. A similar conclusion was reached in S. Shankar’s study [41].

### 3.3. Field Application Evaluation of PBAT and PBAT/UVA Films

#### 3.3.1. Analysis of Functional Period of Films

Table 5 shows the days of the functional period when PBAT and PBAT/UVA films were applied in the field. The functional period of the film was determined by the time when more than 30% of the films in the whole field have a large-area rupture (≥20 cm), and the films could no longer provide the function of increasing soil temperature and preserving soil moisture. In Table 5, it is not difficult to see that the functional periods of PBAT, PBAT/UVA-0C, PBAT/UVA-1C, PBAT/UVA-4C and PBAT/UVA-6C were 40 days, 41 days, 43 days, 45 day and 48 days, respectively. Obviously, the functional period of films that contained UV absorbers was longer than that of pure PBAT. This meant that the introduction of UV absorbers into film could effectively absorb most of the UV light, resulting in slowing down the degradation speed and prolonging the functional period of films. Two main reasons may explain why the PBAT/UVA-6C film exhibited the longest functional period. On the one hand, UVA-6C contained more chromogenic groups that absorb UV rays. On the other hand, UVA-6C combined more closely with PBAT, and its flexible structures brought a stable state to the matrix.

#### 3.3.2. Analysis of Soil Temperature under Films

**Figure 12:**
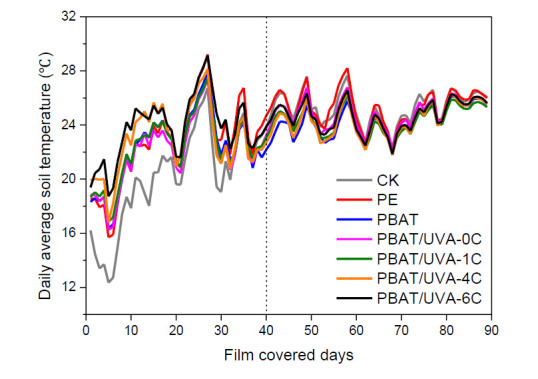
Soil temperature curves under PBAT and PBAT/UVA films.

The variation curves of soil temperature during the whole growth period of maize are shown in Figure 12, and Table 5 shows the cumulative soil temperature in the first 20 and 40 days under the films. The soil temperature was closely related to the growth and development of the crop. In the first 40 days, the average cumulative soil temperature of PBAT/UVA-0C, PBAT/UVA-1C PBAT/UVA-4C and PBAT/UVA-6C films was 0.6 °C, 3.8 °C, 28.0 °C and 61.1 °C higher than the average soil temperature of PBAT. Notably, the average soil temperature of PBAT/UVA-4C and PBAT/UVA-6C even exceeded PE by 10.6 °C and 44.2 °C, respectively. The working principle of UV absorbers is to absorb UV light and release harmless energy such as heat, which was able to supplement the soil temperature. Particularly when the outside temperature was low, the appropriate replenishment of the soil temperature significantly promotes crop growth. Considering that the soil temperature under PBAT/UVA-0C, PBAT/UVA-1C PBAT/UVA-4C and PBAT/UVA-6C films increased by 1.2 °C, 9.8 °C, 31.0 °C and 41.9 °C when compared with PE in the first 20 days, this indicating the good capability of converting UV light into heat by introducing UVA absorbers in the PBAT, and it is more obvious in UVA-4C and UVA-6C. After 40 days, a large-area crack appeared on the surface of the films, resulting in bare soil. Therefore, the subsequent soil temperature changes were mainly from the weather change, and the soil temperature changes between treatments were almost the same, consistent with the results of W. Zhang [42].

#### 3.3.3. Analysis of Maize Yield under Films

The yield information of maize is presented in Table 5. The fresh yield of all mulching films was significantly higher than that of CK, which reflected the role of mulching film in increasing yield Compared with pure PBAT, maize yield under PE, PBAT/UVA-0C, PBAT/UVA-1C, PBAT/UVA-4C and PBAT/UVA-6C films increased markedly by 11.0%, 4.5%, 4.4%, 7.3% and 7.8%, respectively. Furthermore, there was no obvious difference between PE, PBAT/UVA-4C and PBAT/UVA-6C. These data indicate that the existence of UVA in the PBAT showed the advantage of increasing production over PBAT, especially for UVA-4C and UVA-6C. More importantly, the yield-increasing effect of PBAT/UVA-4C and PBAT/UVA-6C was comparable with PE. As mentioned in Section 3.3.2, the increase in production may be related to the rise in soil temperature. Overall, fine light and heat resources were indispensable parameters for promoting crop growth and development.

## 4. Discussions

The three UV absorbers, UVA-1C, UVA-4C and UVA-6C, were prepared by the organic total synthesis in this research. There were certain differences in the structural design of the three UV absorbers. The increase in molecular weight, conjugate structures, and the introduction of flexible alkyl chains with different lengths were considered to enhance the ability of UVAs to absorb UV light and use stability. Compared with UVA-1C (Mw: 346), UVA-4C (Mw: 580) and UVA-6C (Mw: 636) expanded the molecular weight and contained more conjugated structures (one more benzene ring). According to UV-Vis, the order of UV absorption intensity was UVA-6C > UVA-4C > UVA-1C. It was confirmed that a larger molecular weight and more conjugated groups can enhance the absorption intensity of UV light. Besides, in the UVA-1C structure, there was one carbon in the alkyl chain between the benzene ring and the seven-membered ring on the left, while there were four carbons and six carbons in the same position in UVA-4C and UVA-6C, respectively. The different alkyl chains in the UV absorbers structures were used to evaluate their binding degree with polymer materials. From the AFM, the values of surface roughness of PBAT/UVA-1C, PBAT/UVA-4C and PBAT/UVA-6C films were 11.5 nm, 10.8 nm and 9.5 nm, respectively. The smaller the values, the smoother the materials surface, indicating that UVAs were evenly distributed in the PBAT materials. That is, the atomic force between UVAs and the PBAT matrix was strong, and the binding degree between the UVAs and PBAT materials was close. In conclusion, this explained why PBAT/UVA-6C film had the strongest ability to absorb UV and the longest functional period. Since the effect of UVA-6C was so good, this begs the question of whether UVA-8C and UVA-10C have better effects. In many test results of this study, UVA-4C and UVA-6C showed similar performances without leap changes. It can be inferred that UVA-8C may also show an effect close to UVA-6C. In addition, with the expansion of the molecular structure, the difficulty and cost of synthesis increased. This likely indicated that UVA-6C was already the relatively best choice.

## 5. Conclusions

A novel class of ultraviolet absorbers (UVAs: UVA-1C, UVA-4C and UVA-6C) was successfully synthesized, which was confirmed by HRMS, 1H NMR and FTIR. The PBAT/UVA composite films were then fabricated by extrusion blow molding. UVAs were introduced into the PBAT matrix, which endowed PBAT/UVA films with a stronger ability to absorb UV light and prolong the functional period of films compared to pure PBAT. Due to the low migration and volatility of UVA-6C in the film, the validity period of PBAT/UVA-6C in the Xenon lamp aging machine was 240 h longer than pure PBAT and 120 h longer than PBAT/UVA-0C and PBAT/UVA-1C. Compared with PBAT, the functional period of PBAT/UVA-0C, PBAT/UVA-1C, PBAT/UVA-4C and PBAT/UVA-6C in the field application test was increased by 1 day, 3 days, 5 days and 8 days, respectively. Besides, the maize yield under the PBAT/UVA-6C film was significantly increased by 7.8% compared to that under the PBAT film, which is comparable with PE film. This research provided a promising strategy to design a novel high-performance UV absorber, which could be used to fabricate biodegradable plastic films with a longer functional period.

## Figures and Tables

**Table 1:** Composition of PBAT/UVA films.

Sample Films	Component (phr)				
	PBAT	UVA-0C	UVA-1C	UVA-4C	UVA-6C
PBAT	100	/	/	/	/
PBAT/UVA-0C	100	0.1	/	/	/
PBAT/UVA-1C	100	/	0.1	/	/
PBAT/UVA-4C	100	/	/	0.1	/
PBAT/UVA-6C	100	/	/	/	0.1

**Table 2:** Degradation of PBAT and PBAT/UVA films at different times.

Samples	0 h	240 h	360 h	480 h	600 h
PBAT	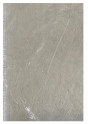	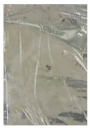	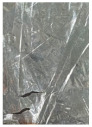	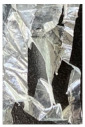	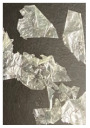
PBAT/UVA-0C	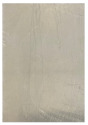	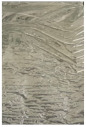	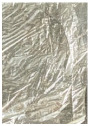	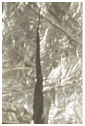	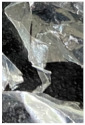
PBAT/UVA-1C	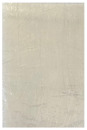	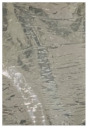	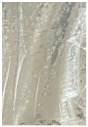	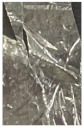	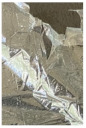
PBAT/UVA-4C	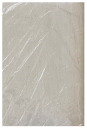	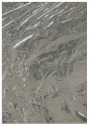	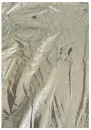	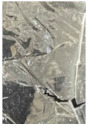	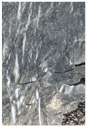
PBAT/UVA-6C	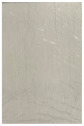	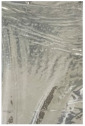	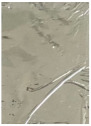	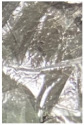	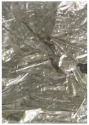

**Table 3:** Classification of degradation grade of films in xenon weatherometer.

Samples	0 h	240 h	360 h	480 h	600 h
PBAT	0	1	2	3	4
PBAT/UVA-0C	0	0	1	2	3
PBAT/UVA-1C	0	0	1	2	3
PBAT/UVA-4C	0	0	0	1	2
PBAT/UVA-6C	0	0	0	1	2

Notes: Grade 0: Grade 1: Cracks or holes < 2 cm; Grade 2: Cracks or holes > 2 cm; Grade 3: Large area fracture; Grade 4: Break into pieces [34].

**Table 4:** GPC analysis of PBAT and PBAT/UVA films.

Samples	Aging Time (h)	Mn	Mw	PDI	Decline Degree of Mn (%)
PBAT	0	29,744	93,227	3.13	/
	240	9906	43,272	4.37	66.7
	360	6040	61,603	5.23	79.7
	480	2596	22,142	8.53	91.3
PBAT/UVA-0C	0	41,884	90,455	2.16	/
	240	18,885	75,473	4.00	54.9
	360	15,834	33,525	2.12	62.2
	480	4984	32,382	6.50	88.1
PBAT/UVA-1C	0	29,632	94,698	3.20	/
	240	19,080	50,429	2.64	35.6
	360	5562	35,804	6.44	81.2
	480	5251	46,163	8.79	82.3
PBAT/UVA-4C	0	26,340	94,331	3.58	/
	240	19,958	69,777	3.50	24.2
	360	10,743	38,889	3.62	59.2
	480	9412	27,666	2.94	64.3
PBAT/UVA-6C	0	36,822	94,444	2.56	/
	240	30,075	76,121	2.53	18.3
	360	15,607	42,280	2.71	57.6
	480	13,701	25,878	1.89	62.8

**Table 5:** Field application data for PBAT and PBAT/UVA films.

Samples	Functional Period (Days)	Accumulated Soil Temperature in the First 20 and 40 Days (°C)	Maize Fresh Weight (kg/hm^2^)
CK (without film)	/	354.0 ± 7.9/806.2 ± 21.6	18,977.1 ± 1273.9 a
PE	/	414.1 ± 7.1/905.9 ± 13.0	24,590.3 ± 390.2 d
PBAT	40	409.8 ± 3.6/888.5 ± 8.8	21,881.9 ± 731.9 b
PBAT/UVA-0C	41	415.3 ± 20.2/889.1 ± 31.6	22,915.7 ± 285.8 bc
PBAT/UVA-1C	43	423.9 ± 6.1/892.3 ± 27.8	22,883.3 ± 449.7 bc
PBAT/UVA-4C	45	445.1 ± 8.2/916.5 ± 17.0	23,601.9 ± 153.7 cd
PBAT/UVA-6C	48	456.0 ± 12.2/950.1 ± 26.2	23,731.7 ± 285.8 cd

(Note: The lower-case letter in the figure shows a significant difference at the level of *p* < 0.05, Duncan’s new multiple range test).

## Supplementary Materials

The following are available online at https://www.mdpi.com/article/10.3390/polym14071434/s1, Figure S1: HRMS analysis of UVAs, Figure S2: ^1^H NMR analysis of UVAs.
