# Supplementary material for: Synthesis of Novel Ultraviolet Absorbers and Preparation and Field Application of Anti-Ultraviolet Aging PBAT/UVA Films
Source: Polymers (Basel). 2022 Mar 31;14(7):1434. doi: 10.3390/polym14071434 (PMC9003559; doi:10.3390/polym14071434)
Supplement: Supplementary file 1 [file polymers-14-01434-s001.zip › polymers-1595286-supplementary.pdf]

polymers

MDPI

Supplementary Materials

Figure S1. HRMS analysis of UVAs.

Figure S2. ¹H NMR analysis of UVAs.

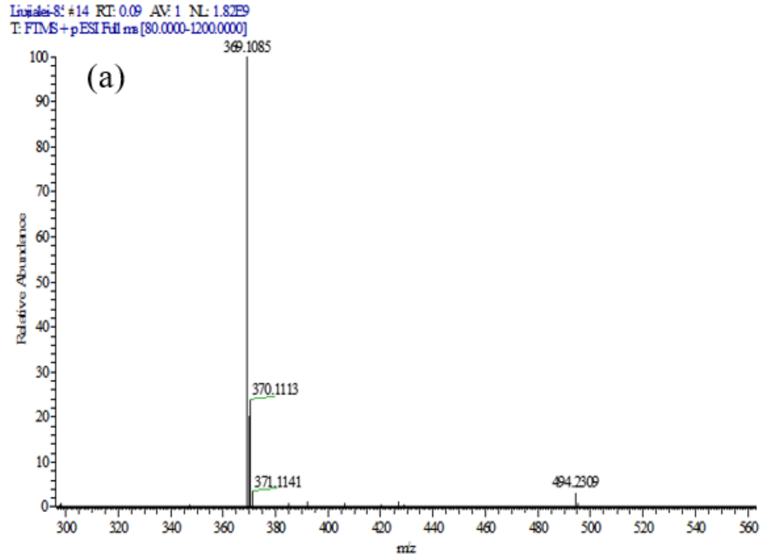

HRMS [M + Na] $^+$ calcd. for $C_{22}H_{18}O_4^+$ 369.1076; found: 369.1085.

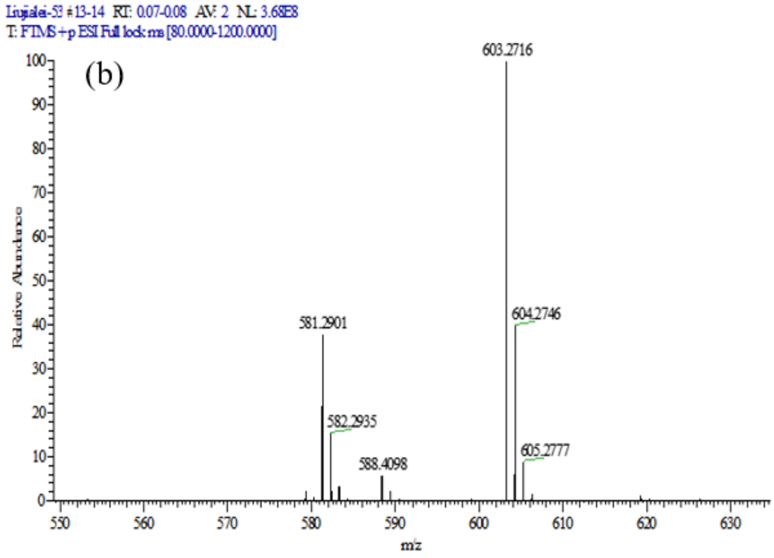

HRMS [M + Na] $^+$ calcd. for $C_{37}H_{40}O_6^+$ 603.2722; found: 603.2716.

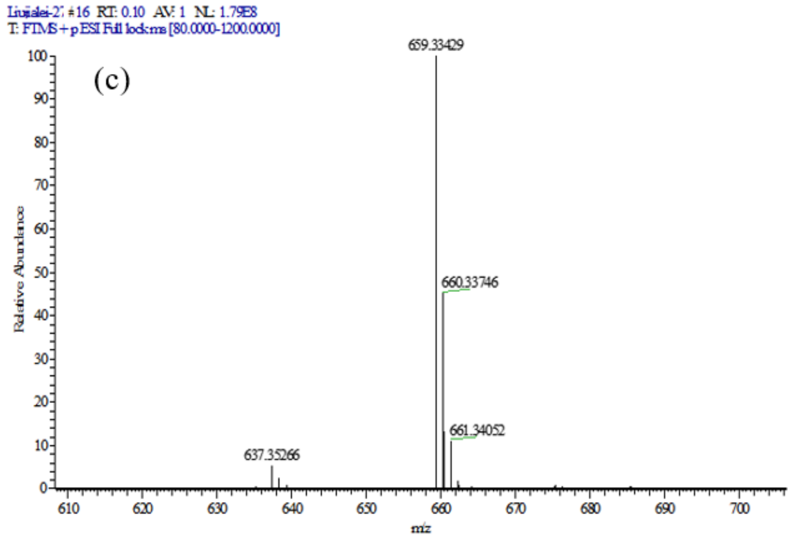

HRMS [M + Na] + calcd. for $C_{41}H_{48}O_6^+$ 659.3330; found: 659.3343.

Figure S1. HRMS spectrum of UVA ((**a**) UVA-1C, (**b**) UVA-4C and (**c**) UVA-6C).

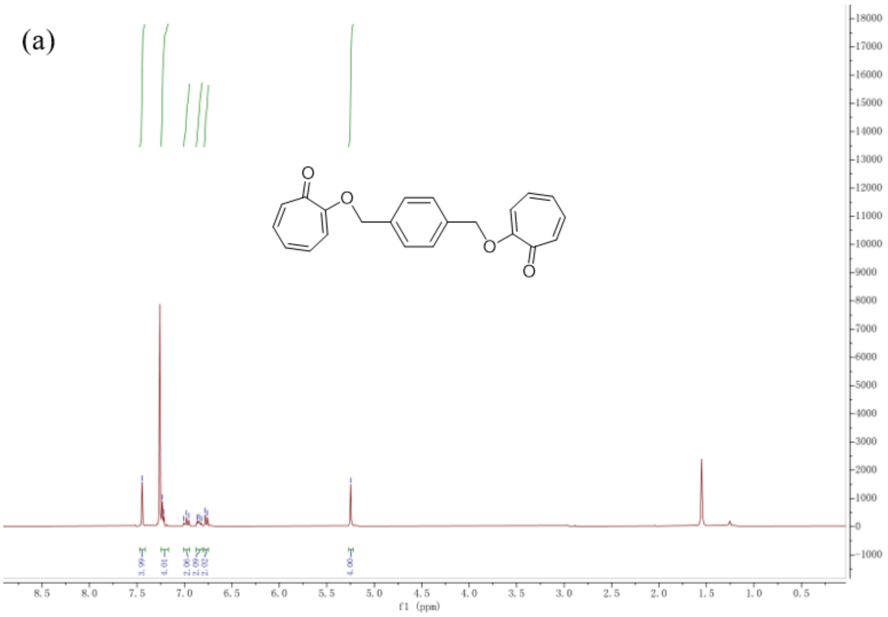

^{1}H NMR (400 MHz, $CDCl_3$, ppm): δ 7.45 (s, 4H), 7.22-7.23 (m, 4H), 6.98 (t, J=8.0 Hz, 2H), 6.85 (t, J=12.0 Hz, 2H), 6.77 (d, J=8.0 Hz, 2H), 5.25 (s, 4H).

(b)

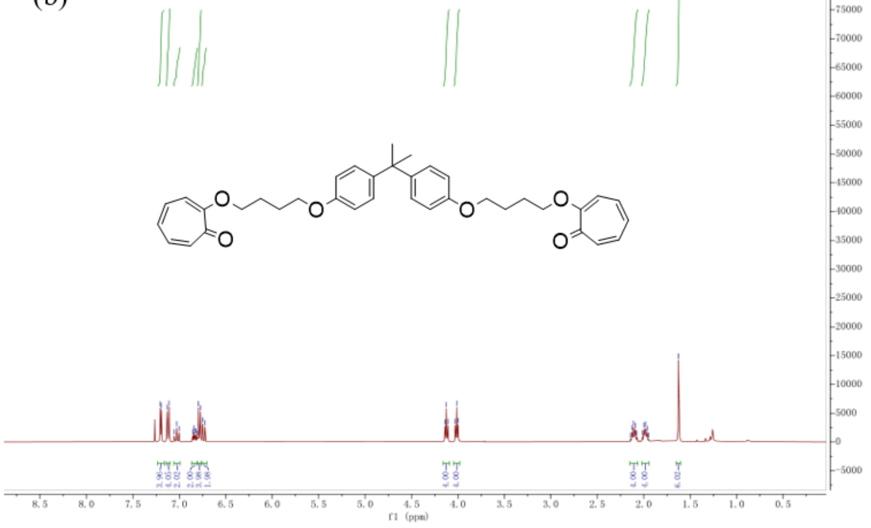

^{1}H NMR (400 MHz, $CDCl_3$, ppm): δ 7.20 (d, J=8.0 Hz, 4H), 7.12 (d, J=8.0 Hz, 4H), 7.03 (t, J= 8.0 Hz, 2H), 6.86-6.81(m, 2H), 6.78 (d, J=8.0 Hz, 4H), 6.73 (d, J=12.0 Hz, 2H), 4.13 (t, J= 8.0 Hz, 4H), 4.01 (t, J=8.0 Hz, 4H), 2.14-2.07 (m, 4H), 2.01-1.95 (m, 4H), 1.63 (s, 6H).

(c)

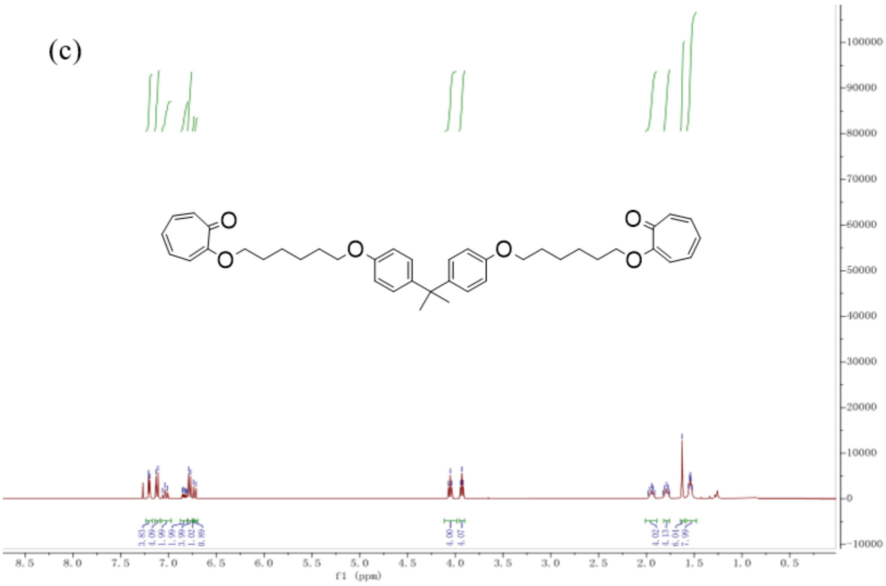

^{1}H NMR (400 MHz, $CDCl_3$, ppm): δ 7.20 (d, J=4.0 Hz, 4H), 7.12 (d, J=8.0 Hz, 4H), 7.03 (t, J= 12.0 Hz, 2H), 6.85-6.80 (m, 2H), 6.78 (d, J=8.0 Hz, 4H), 6.74 (s, 1H), 6.71 (s, 1H), 4.05 (t, J=8.0 Hz, 4H), 3.93 (t, J=8.0 Hz, 4H) 1.98-1.91 (m, 4H), 1.82-1.76 (m, 4H), 1.63 (s, 6H), 1.56-1.52 (m, 8H).

Figure S2. ^{1}H NMR spectrum of UVA ((**a**) UVA-1C, (**b**) UVA-4C and (**c**) UVA-6C).
